# Age of the Late Dr. Mütter

**Published:** 1860-03

**Authors:** 


AGE OF THE LATE DR. MUTTER.—The age of a distinguished man is sometimes a matter of greater importance than the world at large is willing to admit; at all events, it is frequently of considerable interest. The age of Dr. Mütter is stated, by Professor Pancoast, in his late eulogy, at forty-eight. In conversation recently with a physician, himself a gentleman of distinction, he assured us that he had known Dr. Mütter from his earliest boyhood, and that his age, at the time of his death, could not have been less than fifty-six. We remember when Dr. Goodman died his age was stated at thirty-two or three, but was subsequently ascertained to have been thirty-seven. The appearance of Mütter certainly denoted a more advanced age even than that assigned to him by his friend and early companion.

